# Using Standardized Test Scores to Include General Cognitive Ability in Education Research and Policy

**DOI:** 10.3390/jintelligence6030037

**Published:** 2018-08-02

**Authors:** Jonathan Wai, Matt I. Brown, Christopher F. Chabris

**Affiliations:** 1 Department of Education Reform, University of Arkansas, Fayetteville, AR 72701, USA; 2 Autism & Developmental Medicine Institute, Geisinger Health System, Lewisburg, PA 17837, USA; mibrown1@geisinger.edu (M.I.B.); chabris@gmail.com (C.F.C.); 3 Institute for Advanced Study in Toulouse, 31015 Toulouse, France

**Keywords:** education policy, general intelligence, SAT, college rankings, elite schools

## Abstract

In education research and education policy, much attention is paid to schools, curricula, and teachers, but little attention is paid to the characteristics of students. Differences in general cognitive ability (*g*) are often overlooked as a source of important variance among schools and in outcomes among students within schools. Standardized test scores such as the SAT and ACT are reasonably good proxies for *g* and are available for most incoming college students. Though the idea of *g* being important in education is quite old, we present contemporary evidence that colleges and universities in the United States vary considerably in the average cognitive ability of their students, which correlates strongly with other methods (including international methods) of ranking colleges. We also show that these *g* differences are reflected in the extent to which graduates of colleges are represented in various high-status and high-income occupations. Finally, we show how including individual-level measures of cognitive ability can substantially increase the statistical power of experiments designed to measure educational treatment effects. We conclude that education policy researchers should give more consideration to the concept of individual differences in cognitive ability as well as other factors.

## 1. Introduction

In education research and education policy, there is a great deal of attention paid to teachers and schools, but very little attention paid to student characteristics. Chief among these ignored characteristics is intelligence or cognitive ability (often known as IQ). This is a problem because whenever attempting to determine the impact of schools or teachers, one must first account for what is most likely the largest source of variance: the cognitive abilities that students bring to the classroom. In this paper we show that general intelligence is an overlooked variable in education and policy research, that when fully considered and accounted for, can reorient the way current education literatures are interpreted, improve the way future educational studies are conducted, and more rapidly lead to effective solutions to help educate children.

The idea that there is significant overlap between ability and achievement tests and that cognitive abilities are important in education is not new. Almost a century ago, Kelly ([1], p. 64) introduced the idea of the “jangle fallacy” as “the use of two separate words or expressions covering in fact the same basic situation, but sounding different, as though they were in truth different” (c.f., [2], p. 347). In introducing the jangle fallacy, Kelly [1] was referring to the significant overlap of the traits measured by group intelligence tests and school achievement tests. Many other scholars have also pointed out the importance of general intelligence in education (e.g., [2,3,4,5,6]).

### 1.1. What Does the SAT Measure?

The Scholastic Assessment Test (SAT) and American College Test (ACT) are two examples of standardized tests widely used in college admissions. Perhaps because of the high stakes nature of these exams and the fact they are administered to over a million students every year, they have come under fire despite their considerable psychometric reliability and validity. One of the most common critiques of the SAT is that it is nothing more than a “wealth test” [7]. It has been argued that the verbal section of the SAT measures “the size of student houses” [8] and that “the only thing the SAT predicts well now is socioeconomic status” [9]. However, these statements are contradicted by the fact that the SAT and ACT both actually measure general intelligence or *g* to a large degree [10,11], see also [12], despite the test companies themselves marketing them as academic or achievement measures. For example, Frey and Detterman [10] showed the correlation between *g* derived from the Armed Services Vocational Aptitude Battery (ASVAB) and the SAT in a sample of 917 participants was 0.82, and Koenig et al. [11] showed the correlation between *g* derived from the ASVAB and the ACT in a sample of 1075 participants was 0.77. SAT scores are also correlated between 0.75 to 0.86 with GRE scores (see [13,14], respectively), which is additional evidence suggesting that the SAT measures something stable, like general ability or IQ. As Hunt ([15], p. 157) pointed out, “College entrance is substantially a cognitive screening process.” In fact, Kelley [1] noted that the overlap between intelligence tests and school achievement tests was about 90% and in their independent analysis Coleman and Cureton [2] noted that this overlap was about 95%. Thus, this overlap has been noted for many decades.

Such statements are also contradicted by the broad research evidence showing the predictive validity of all kinds of standardized achievement tests on important academic and career outcomes (e.g., [16]), and the effects of cognitive ability on life outcomes are stronger than the effects of wealth or SES [17]. The fact that the SAT and ACT measure *g* in part, serve as reasonable proxies for *g*, and that many researchers, the test companies themselves, and the public do not understand and accept this, is a core reason myths such as the idea that these tests are “wealth tests” continue to persist (e.g., see “Why the new SAT scores are meaningless” in *The Washington Post* by Strauss [18]). Counter to claims of such tests being “biased,” low income talent has been shown to be underperforming relative to their ability level [19], and could be identified more fairly by “universal screening” of all students [20,21,22,23].

One class of variables that are directly relevant to the field of education broadly is the structure of cognitive abilities, specifically general intelligence or *g* [24,25,26,27]. Spearman [28] classically argued that the specific content of mental tests was not all that important because *g* enters into the performance on any mental test. Research has corroborated his initial claim, showing that *g* is measured to some extent by nearly any challenging cognitive test with a variety of question types and tasks, independent of analytic technique or items used [26,29], and even when tests are initially designed to measure a variety of achievements and abilities, *g* is found (e.g., [30,31]). Cognitive *g* and academic achievement *g* are roughly the same from a measurement perspective [32], meaning the variance accounted for by *g* among cognitive ability tests is roughly the same variance as that accounted for by *g* among academic achievement tests. The SAT and ACT are often thought of as achievement and/or aptitude tests but as indicated earlier, are in fact reasonable proxies for *g* [10,11,12].

### 1.2. What Is the Impact of Teachers and Classrooms after Accounting for g?

If one accepts that many standardized tests used throughout education measure *g* to a large degree, this means that student abilities are actually one of the most important sources of variance to account for in educational contexts. Detterman ([25], p. 9) reviewed a large body of literature taking student abilities into account in addition to teacher and school impacts, concluding that “schools and teachers account for less than 10% of the total variance in academic achievement and that student characteristics account for 90%. This observation has been supported by many studies and reviews and has been known at least since the 1960s. In fact, in the few studies that estimate the variance in academic achievement attributable to teachers not confounded with schools it is probably only 1–8%. It should also be noted that though this is a small amount of the total variance, teacher effects on school achievement are probably the largest component of within school factors when student characteristics are ignored.” Prominent studies have suggested that kindergarten classrooms affect earnings at age 27, without acknowledging the large literature suggesting the potential role of general ability in later academic and economic achievement (e.g., [33]), and there is also a long history of using standardized tests to measure the value added by teachers [34,35]. In both cases not discussing the importance of *g* in academic and occupational achievement confounds the impact of such findings. This is not to say that teachers or classrooms are unimportant, only that cognitive abilities of students need to be accounted for to fully understand the impact of teachers and classrooms and ways to most effectively help students.

### 1.3. The Fallacy of the Neglected Aspect

There has been a recent surge in the social sciences and other fields to take action to improve research practices, spurred on by numerous high profile critiques of the way research has traditionally been conducted (e.g., [36,37,38]), with much focus on a lack of reproducibility and how to improve that, including specifically in education research [39]. Schmidt [40] noted that there are other, potentially more fundamentally important, problems with building a cumulative social science, including the role of “omitted relevant research.” Other scholars have termed this the “fallacy of the neglected aspect,” [41]. Accounting for all of the current scientific evidence at any given time (e.g., [42,43]) is quite difficult, but we argue here that established and obvious variables like *g* should be accounted for as a good first step. Given that governments often base social programs and education policies around studies conducted within the field of education, what becomes taken as the gold standard as the basis for policies is incredibly important. We make the case in this paper that accounting for general ability in education research and policy is critical to help advance the field. Though this idea is not new, it is remarkable that the large body of evidence that has accumulated across many decades remains unaccounted for in much research and practice in education and policy. Therefore, we provide contemporary data to build on this longstanding chain of research evidence in the hope that education research and policymakers might account for this cumulative evidence.

Using this line of research by Detterman and colleagues [10,11] provides a way to study the role of general ability indirectly through education. In this paper we use two studies to provide examples of how SAT, ACT, and other test scores as measures of general ability helps us think differently about research in education, but also more broadly across the social sciences. We conclude that because of the widespread use of SAT, ACT, and other standardized tests in education, there are many novel ways to (indirectly) examine the role of general (or even specific) abilities in education research and policy. Accounting for the fact that many standardized tests are measures of cognitive abilities can help reorient our thinking about literatures that directly bear on education, and provide a starting point from which more solid education policies can be built to more effectively help children.

Study 1 examines how colleges and universities are distributed by general ability—a higher education “*g* vector”—and how much overlap there is across this wide range of institutions. Study 2 examines how using this *g* vector in part can help study the reasoning abilities of various other groups, such as highly select occupations and leadership positions.

## 2. Study 1: A Higher Education “*g* Vector”: Colleges Distributed by General Ability

### 2.1. Sample 1: U.S. News & World Report SAT and ACT Scores for Colleges and Universities

The first author collected data from the *U.S. News & World Report* college rankings in 2014 [44] by recording the 25th and 75th percentile SAT and ACT scores reported to *U.S. News* from four core lists: national universities, national liberal arts colleges, regional universities, and regional colleges. All the ACT (composite) scores were first translated to SAT (Math + Verbal) scores using a concordance [45], and then an average of the 25th and 75th percentile scores was taken to indicate an overall general reasoning score for each school. Only 5 schools reported an average score alone thus 25th and 75th percentile scores were not available. The total sample included 1339 schools (see Supplementary A), where we also recorded the *U.S. News* National University Rank, *U.S. News* Liberal Arts Rank, a “revealed preference ranking” ([46], p. 425), a ranking based on students’ revealed preferences: “the colleges students prefer when they can choose among them,” and the *Times Higher Education* “World University Rankings” and “U.S. College Rankings” [47]. By linking the *Times Higher Education* World University Rankings with average SAT scores we sought to create a conversion table and corresponding equation for researchers who might want to estimate the average SAT or general ability level of a non-U.S. institution for research purposes. As validations of SAT scores as measures of *g* we also included measures of cognitive performance that might be caused by indicators of *g*, specifically rankings of the brainpower of students made by the company Lumosity using their brain games [48], and Freshman critical thinking performance on the Collegiate Learning Assessment Plus (CLA+; https://graphics.wsj.com/table/THINKTEST_0510; [49]). The CLA+ data compiled by *The Wall Street Journal* examined the extent to which “critical thinking skills” were improved from Freshman to Senior year and the test was administered at these two time points. We used the Freshman scores for our analysis because this was the first exposure to the test, which is likely a better indicator of ability than the latter exposure.

### 2.2. Results

**Figure 1:**
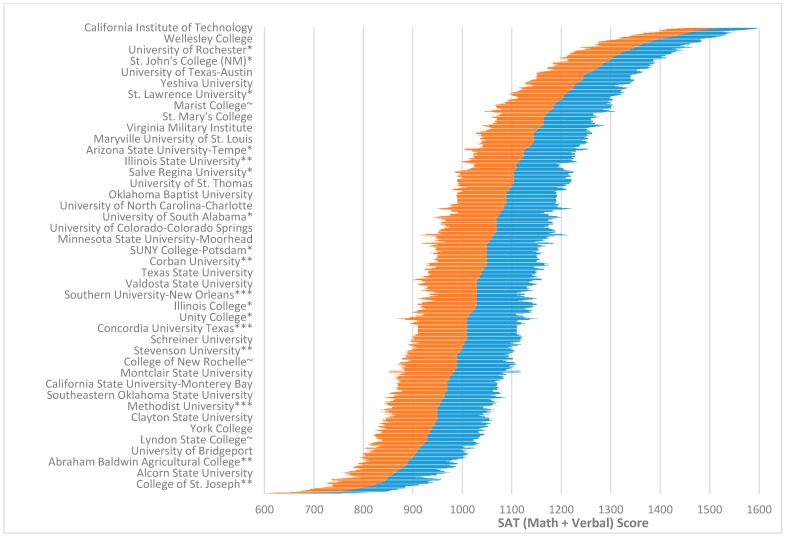
Overall distribution of 25th to 75th SAT (Math + Verbal) percentile scores for colleges and universities included in this study (for a full list see Supplementary A). 25th percentile scores for an institution are indicated by the far left point of the left region (**orange**) of the graph, whereas 75th percentile scores are indicated by the far right point of the right region (**blue**). The dividing line between the two sides is roughly the 50th percentile (the average between the 25th and 75th percentile scores). Selected school names are highlighted along the y-axis for ease of reading. The full list of schools used to create Figure 1 can be found in Supplementary A. * At least some students were not required to supply scores to the school; ** The school did not report all students it had scores for, or did not tell *U.S. News* if it had; *** The data was reported to *U.S. News* from a previous year; ~ The school may not require scores from all applicants and may not have submitted data for all students.

Figure 1 (see full data in Supplementary A) illustrate a higher education “*g* vector.” Figure 1 shows all 1339 schools to illuminate the full structure of the data indicating there is an incredible range between the school with the highest average SAT (Math + Verbal) scores (California Institute of Technology: 25th percentile = 1490; average = 1545; 75th percentile = 1600) and the school with the lowest average scores (Livingstone College: 25th = 530; average = 660; 75th = 790). This translates to a range of 940 points (math and critical reading combined). Given the mean and SD for each individual SAT subtest is roughly 500 and 100 respectively, this is about a 4.7 SD difference (Shaw et al. [50] estimate a combined SD of 201). Figure 1 and Supplementary A indicate that the 75th percentile of students at the California Institute of Technology is already at 1600 which is the maximum of the scale, suggesting a headroom problem on the SAT and ACT for students at the most select colleges (this means a fourth of Caltech students could score above the max score if the test was harder and designed to measure higher performance). There is also a large amount of overlap across schools between the scores of 900 and 1300. This aligns with data from the College Board [51] which estimates based on their norm table that 66% of people who take the SAT score between 900 and 1300. Supplementary A also shows the discrepancy between average SAT and ACT scores of a school and the respective *U.S. News* ranking of that school.

To determine how average SAT (M + V) of a school relates to other rankings, an analysis was conducted comparing average ability scores of a school in relation to *U.S. News* National University Rank, *U.S. News* Liberal Arts Rank, and the “revealed preference ranking” [46]. These respective alternative rankings are all included in Supplementary A. Both the *U.S. News* National University Rank (N = 202, r = −0.892, *p* < 0.001) and *U.S. News* Liberal Arts Rank (N = 181, r = −0.890, *p* < 0.001) were highly correlated, *Times Higher Education* U.S. Rank (N = 1008, r = −0.787, *p <* 0.001) and the revealed preference ranking were slightly less correlated (N = 109, r = −0.757, *p* < 0.001). Average SAT was least correlated with the *Times Higher Education* World Rank (N = 158, r = −0.659, *p* < 0.001). These correlations with other rankings suggest that general ability accounts for a large part of the variance in alternative rankings alone (ranging from 43.4% to 79.6%). To further validate average SAT (M + V) as a measure of ability and to examine an alternative measure of *g* at the level of colleges and universities we found that the SAT and Lumosity rankings were highly correlated (N = 447, r = −0.794, *p* < 0.001). This provides evidence suggesting that though Lumosity games are unlikely to improve brainpower [52], they are in fact reasonable measures of brainpower or *g* even at the aggregate level of institutions of higher education. Additionally, we examined the correlation between SAT scores and Freshman critical thinking test scores on the CLA+, which were very highly correlated (N = 68, r = 0.846, *p* < 0.001). Both of these findings on “brain games” and a “critical thinking measure” align with Spearman’s [28] idea of the “indifference of the indicator” given these were measures not designed or intended to measure *g*, but likely do in some capacity.

Table 1 is a conversion table that can be used to interpolate average SATs for non-U.S. schools. It was developed by examining the 158 U.S. schools with reported average SATs that made it onto the *Times Higher Education* World Rank and taking an average of the SATs for each band of schools in the World Rank. We also include an equation here based on linear regression (R squared = 0.43, F(1156) = 119.6, *p* < 0.01): predicted SAT (Math + Verbal) = 1351.7076 + (−0.4419 * World Rank). Table 1 and/or the corresponding equation could be used to estimate the SATs or general ability level of students at non-U.S. schools by looking up those schools in Supplementary B. To give an example, the list of non-U.S. schools that would make it within the top 25 on our general ability ranking (average 1406 from Table 1) are University of Oxford, University of Cambridge, Imperial College London, ETH Zurich—Swiss Federal Institute of Technology Zurich, UCL, National University of Singapore, University of Toronto, and the London School of Economics and Political Science. All of these are highly selective schools. Supplementary B includes all schools listed on the *Times Higher Education* world ranking in 2018 so that researchers can use this along with Table 1 for interpolation purposes.

## 3. Study 2: College Attendance as a Proxy for General Ability to Study Other Groups

### 3.1. Sample 2: U.S. Occupationally Select Groups and the SAT and ACT Scores of the Colleges and Universities They Attended

Summary data (total N = 11,745) were taken from multiple prior publications [53,54,55,56,57]. The summary data was restricted to just U.S. groups. The final sample included *Forbes* billionaires (N = 424), Wealth-X 30-millionaires (N = 8649), Fortune 500 CEOs (N = 500), World Economic Forum in Davos attendees (N = 661), active federal judges (N = 789), House members (N = 441), Senators (N = 100), *The New Republic* masthead (N = 95), and the most powerful men (N = 27) and women (N = 59) according to *Forbes*. What follows are brief descriptions of groups included that readers may be less familiar with.

People invited to the World Economic Forum in Davos, Switzerland are among the political, business, academic, and other leadership of society. The men and women identified as “most powerful” by *Forbes* were selected based on their power and influence and these two groups included many politicians and business leaders. Wealth-X is a company that collects data on individuals with net worth’s that are at or above $30 million USD. *The New Republic* is an elite and influential magazine which was used because the data were publicly available.

By selecting on the elite group of interest, this study composes a retrospective study approach that can be used to examine the importance of general intelligence in developing occupational expertise. Though one would expect some relationship between test scores and top colleges given they are used for admission, the extent to which there is a connection between such test scores and elite occupations as well as the variation one might observe across elite occupations remains to be empirically determined. As tests such as the SAT and ACT are almost uniformly required for college admission in the U.S. and measure *g* to a significant degree [10,11], information where individuals attended for undergraduate was used as an indicator for general intelligence [58]. Additionally, graduate admissions tests such as the Graduate Management Admissions Test (GMAT), Law School Admissions Test (LSAT), and Graduate Record Examination (GRE) all measure similar constructs as the SAT and ACT but provide an ability measure at a different stage in the higher education process allowing a wider net to be cast to identify high ability individuals, thus information where individuals attended for graduate school was also used as an indicator for general ability. Attendance at colleges and universities that indicated average standardized test scores on the combined SAT Math and Verbal subtests (or ACT equivalent) or on graduate admissions tests such as the LSAT, GMAT, and GRE were used to specify the percentage of individuals from each group likely in the top 1% of cognitive ability and who attended an “Elite School” as defined for this study. In addition, the percentage independent of this top 1% that attended graduate school, college, or did not report or did not attend college was also examined. For more detail regarding the method, including a list of the colleges and universities that had average test scores in the top 1%, see the methods section, limitations section, and Table 1 of Wai [54]. This method essentially takes schools from the top end of the higher education *g* vector discussed in Study 1 of this paper.

### 3.2. Results

**Figure 2:**
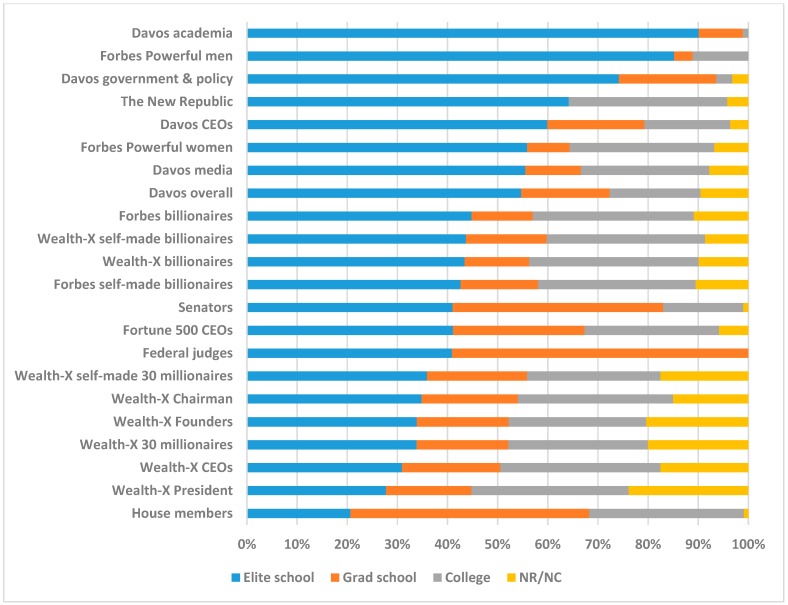
Elite school attendance and general ability level of U.S. occupationally select groups. Blue bars: The percentage of each occupationally selective U.S. group that attended an “Elite school” (undergraduate or graduate) and were in the top 1% of general cognitive ability. Orange bars: The percentage that attended a “Grad school” independent of the Elite school category. Gray bars: The percentage attending “College” independent of the Grad school and Elite school categories. Yellow bars: The percentage that did “not report” or had “no college.” Elite school + Grad school + College + NR/NC sum to 100%. Data were taken from multiple research papers and adapted for this figure focused on the U.S. [53,54,56,57].

Figure 2 shows the fraction of each occupationally elite group in the top 1% of general cognitive ability as indicated by test scores. Specifically, the “Elite School” category indicates the percentage of each occupation that attended one of the schools (either at the undergraduate or graduate level) with average test scores that put them in the top 1% of ability. The “Graduate School” category indicates the percentage of each occupation that attended graduate school but not an elite graduate school so is independent of the Elite School category. This group is likely to be in the top percentiles of cognitive ability. The “College” category indicates the percentage that attended college in some capacity but not a Graduate School or an Elite School. The “NR/NC” category indicates the left over percentage that did “not report” any schools attended or had “no college.” These independent categories sum to 100%.

Figure 2 shows that about 50% of U.S. leaders attended an elite school and were in the top 1% of cognitive ability. Given people in the top 1% should be expected to be represented at that rate for the general population this shows that top 1% people are overrepresented in these elite occupations by a factor of about 50. House members attended elite schools the least (20.6%) and academics who went to Davos attended elite schools the most (90.1%). Across all occupations studied, 91.1% attended at least college. This ranged from 76.2% for 30-millionaire company presidents (Wealth-X President) up through 100% for Davos academia, federal judges, and *Forbes* most powerful men.

Overall, House members were the least select on education and corresponding brainpower, with selectivity increasing from that point onwards, from 30-millionaire CEOs (Wealth-X CEOs), 30-millionaires overall, federal judges, Fortune 500 CEOs, senators, billionaires overall, Davos attendees overall, Davos media, *Forbes* most powerful women, Davos CEOs, The New Republic, *Forbes* most powerful men, and Davos academia at the pinnacle. Davos attendees, *The New Republic* masthead, selective government officials, selective academics, and heads of countries (from the powerful men and women lists) were in the top half of the education and brainpower distribution. Billionaires were about in the middle, whereas House members, 30-millionaires, federal judges, and Fortune 500 CEOs were in the bottom half of the education and brainpower distribution.

## 4. Limitations

### 4.1. Study 1

This study does not include every college or university in the U.S., only those 1339 schools that reported such data to *U.S. News & World Report* in 2014. Additionally, the only systematic data we had was for the 25th and 75th percentile and average of SAT and ACT scores of one of these schools, which gives us little to no information about the score distributions above and below the middle 50% of students. For example, large state schools, such as University of California-Berkeley, likely have a group of students in the right tail of the distribution (many large state schools have “honors colleges”) which have average SAT or ACT scores quite similar to highly select schools with small populations, such as high ranking liberal arts colleges (see discussion in Pinker [59]). Though the relationship between the median test score of a school and ranking is not necessarily surprising, in this study we are simply trying to describe the differences in test scores and corresponding ability across institutions, not to make any causal statements about institutional rank and cognitive ability.

### 4.2. Study 2

Average standardized test scores of a college or university—at the undergraduate or graduate level—were used as a proxy for general intelligence level. Therefore, the method was not optimal in that individual test scores were not used (as they were not publicly available), and we cannot disentangle cognitive ability from education or other factors. However, average test scores from U.S. schools, in our view, were reasonable estimates for elite occupational group estimates of cognitive ability as there are ways in which school attendance provides both overestimates and underestimates of ability, and these sources of error are likely randomly distributed when considering group estimates. For example, the method may underestimate cognitive ability because very high scorers may not have chosen to attend a top school due to factors like financial limitations, limited scholarships, and a need to stay close to home. Or, the method may overestimate cognitive ability because some students have their chances of admission boosted by being legacies, athletic stars, having political connections, or affirmative action and thus were accepted with below average academic records and/or test scores [60,61,62]. It is reasonable to think factors in both directions are operating. The people who pursue and obtain positions of elite leadership in U.S. society who were part of this study are probably non-representative not only on cognitive ability but also many attributes such as being highly motivated academically but also engaging in deliberate practice, being willing to take risks, and also likely desire status, wealth, and power. Therefore there are many intellectually talented people who are not in one of these occupations studied and have pursued careers at every level and walk of life. This method also does not account for the role of other important factors, such as institutional effects, gender roles, or luck.

## 5. Discussion

Study 1 showed that by using SAT and ACT scores as proxies for general intelligence measures, a higher education *g* vector can be developed. This distribution gives us information on the range and overlap of the students who attend the schools, and also can be used to think about the average ability of students at different schools and the implications of that ability average and range for the level of instruction and, given the link between abilities and educational and occupational outcomes (e.g., [16,63]), expectations for outcomes of students from those schools.

Study 2 took a highly select portion of the schools in Study 1 and used that as a proxy for general intelligence as a way to study the abilities or “*g* loadings” (e.g., [64]) of various highly select occupations or positions of leadership in U.S. society—essentially a measure of how “cognitively complex” such occupations are. Therefore not only is there wide variation in cognitive ability distributed across colleges but also wide variation in cognitive ability distributed across high level occupations.

### 5.1. Standardized Tests Throughout Education as Proxy Measures of g

Though, to our knowledge, specific studies have shown the SAT and ACT are reasonable proxies of *g*, the idea behind Spearman’s [28] indifference of the indicator suggests it is very likely that nearly all standardized mental tests typically used to measure achievement also function by proxy as cognitive ability tests as well. In Study 1, we showed that the correlation between “brain games” and a “critical thinking” measure, which were intended to measure different constructs, in fact ended up likely measuring *g* to a large degree. Belkin [49] lamented that many colleges fail to improve critical thinking skills by examining CLA+ score differences between Freshman and Senior years. Our analysis suggests that critical thinking measures may in fact largely be proxies of *g* just like SAT and ACT scores, and even brain games. Additionally, to illustrate how the correlation between SAT or ACT scores and rankings has remained similar across time we note that other authors [65] have used the 2002 *U.S. News* rankings and examined the correlation between SAT/ACT scores and ranking for the top 50 national universities, finding it was r = −0.89. This is nearly identical to our current analysis using the 2014 *U.S. News* National University rank, where we found it was r = −0.892).

This of course does not mean that *g* is the only dimension measured by high school SAT or ACT scores and other measures. These scores are composed of *g* plus variance specific to that measure, as well as error. Additionally, high school SAT and ACT scores reflect developed abilities which can be impacted by education or other factors earlier in development. SAT and ACT scores are used in talent searches in the U.S., where students at age 12 take one of these tests as part of a talent search to qualify for advanced educational programming, and this data has been used to study the role of specific abilities such as math and verbal ability prediction [66] as well as sex differences and the Flynn effect [67]. Additionally, international comparison tests such as TIMSS, PISA, and PIRLS have been utilized as proxies of IQ or *g* [68]. More broadly, many standardized tests throughout K-12 essentially can function as reasonable general ability proxies. Simply understanding that such tests can function as general ability proxies can help counteract the myths that the SAT is a “wealth test” or that SAT “scores are meaningless.” Additionally, taking into account the larger body of evidence going back to the 1960s suggesting the largest source of variance in student achievement is really student characteristics (of which *g* is a large part; [25]) can help the impact of teachers and schools be more appropriately assessed and interventions to help students to be developed and evaluated.

### 5.2. Cognitive Segregation in Higher Education and Society

Hunt ([15], p. 158) noted that “the personal experiences of most college/university students is restricted to populations that have substantial range restrictions with respect to intelligence,” and provided an example showing that students at University of California-Berkeley and students at California State University-Long Beach, for example, overlapped very little in terms of cognitive ability. Because of this “cognitive segregation,” which is evident in both Study 1 and Study 2 of this paper, many highly select pools of students from elite schools, which by and large are the source of numerous leaders of society as Study 2 indicates, go through education and life without having exposure to students in the full range of cognitive ability. Supplementary A and Figure 1 can give an understanding of when a large proportion of students at a given school do not overlap. For example, the 25th percentile SAT score for Harvard University is 1410, indicating that 75% of Harvard students score at or above this mark. University of North Carolina-Chapel Hill, one of the best and highly selective public institutions, has a 75th percentile SAT score of 1410, indicating that 75% of UNC students score at or below this mark. The majority of students at Harvard and UNC-Chapel Hill do not overlap in terms of general cognitive ability, though 25% do. The same can be said for UNC-Chapel Hill and a school with a much lower middle 50% of scores, and so on throughout the full range of schools. This separation by cognitive ability is already evident in the data we show in this paper, but because students who are admitted to college or university have already been cognitively screened, in the wider society cognitive segregation is an even larger issue. The impact of such insularity in terms of the range of individuals one is exposed to especially in higher education, who they choose to marry and socialize with, and the impact of that on society is something that may be worthwhile to explore in future research.

### 5.3. Standardized Tests, When Used to Test all Students, Improves the Identification of Disadvantaged Students

Researchers have uncovered that testing *all* students on the ACT or SAT—termed “universal screening”—improves postsecondary attainment and can narrow income gaps in college [21,69]. This finding also is uncovered in gifted education, where universal screening of students improves the representation of disadvantaged and underrepresented minority students, in comparison to parent and teacher nominations [20,22,23]. Given that these measures are reasonable proxies for intelligence tests, this indicates that to identify disadvantaged students most effectively, the IQ test is more fair and unbiased than human judgments. Universal screening of students to assess both level and pattern of academic strengths, when coupled with educational programming matched to such strengths, would do much to improve the human capital and life prospects of disadvantaged students.

### 5.4. The Importance of Other Individual Differences Beyond g

Though our focus in this paper is placing emphasis on what we view the weight of evidence suggests is the largest source of variance to account for in education research and policy, this does not take away from the importance of other factors in addition to cognitive abilities. Educational and occupational expertise is multifactorial in nature (e.g., [70]), and other aspects that are important include specific cognitive abilities (e.g., verbal, math, and spatial), personality, interests, motivation, effort, and luck, among others. Individual differences in “motivational profiles” [71] could be important to include in more sophisticated models including cognitive abilities and other factors. Considering motivational profiles, for example, might shed light on the types of students who select into elite schools in Study 1 or in elite occupations and positions of leadership in Study 2. This could ultimately play an important role to understand the full array of influences on individual educational attainment and career achievement beyond the contribution of *g*. Of course, just like cognitive abilities and other individual differences, motivation is heritable to a similar extent [72]. More broadly, individual differences research, and its extensive history across a wide array of variables, are important to consider in education research and policy [73].

### 5.5. Accounting for g in Educational Observational Studies and Interventions

If general intelligence is not included in educational studies and interventions, then causal power may actually be incorrectly attributed to factors that, at least in part, may be caused by *g* itself, such as early childhood achievement. Thus, measures of *g* should be systematically accounted for in observational studies of all kinds. General intelligence measures should also be consistently accounted for in randomized intervention experiments. For example, some variance will be captured that otherwise might be incorrectly attributed to the intervention, as long as a measure of *g* is included in the analysis of a randomized experiment or the researchers stratify the randomization by *g*. Because post-hoc analyses of subgroups (especially in experimental studies) have lower power and smaller samples and are more likely to result in false positive inferences, accounting for *g* becomes even more important. Essentially, given the enormous body of evidence pointing to general intelligence as a core causal predictor, it should be accounted for first in a wide class of educational studies, so that our understanding of the effectiveness of interventions can be more clearly evaluated, understood, and be leveraged to help kids [74].

Not only can accounting for *g* help prevent the incorrect attribution of intervention effects, but it can also boost statistical power. When using *g* as a covariate, the variance in the dependent variable will be reduced by a factor of $\sigma_{e}^{2}\left(1-\rho_{xy}^{2}\right)$ [75]. In a randomized study, the covariate can be assumed to only decrease the error variance in the dependent variable which reduces the standard error for the observed intervention effect [76]. This leads to more precise estimates and allows researchers to detect effects with smaller sample sizes. This increase in power is illustrated in Table 2, where we report the necessary sample size to detect an effect (*d*) based on the relationship between the covariate and the dependent variable (*ρ*). The change in required sample size can be calculated by first determining the necessary sample needed to observe a given effect using a simple *t*-test. This value can then be multiplied by $\sqrt{1-\rho^{2}}$ to estimate the required sample size after including the covariate [77]. In cases where the covariate is expected to be strongly related to the dependent variance (*ρ* = 0.5), including the covariate reduces the required sample size by roughly 25%.

## 6. Conclusions

Due to the widespread use of the SAT, ACT, and other such tests in education, there are many novel ways to examine the role of intelligence in education by proxy. Additionally, the fact that such tests also serve as general ability proxies has wide ranging implications, including understanding the general ability range of students at various colleges as well as the abilities of people in highly selective occupations and how that might impact society. The simple fact of thinking about SAT, ACT or other scores as general reasoning measures should reorient one’s thinking and use of much of the existing educational research and policy literature. Sometimes scientific advance is as simple as bringing established knowledge in one field and exploring its implications in another (e.g., [78]). Exploring these and other implications in education research and policy can help advance the field of education—by taking into account as much of the relevant information as possible—thus increasing the likelihood of improving the understanding of ways to more effectively help children.

## Figures and Tables

**Table 1:** Conversion table to interpolate average SATs for non U.S. schools.

Times Higher Education World Rank	Average SAT (Math + Verbal)	Number of Institutions
1 to 10	1499	7
11 to 25	1406	11
26 to 50	1343	8
51 to 100	1281	17
101 to 150	1326	7
151 to 200	1249	12
201 to 300	1205	21
301 to 400	1182	26
401 to 500	1146	15
501 to 600	1151	14
601 to 1000	1104	20

Note. This conversation table can be used to estimate average SAT (Math + Verbal) scores for non-U.S. schools listed in Supplementary B. Alternatively, one can also use the following equation: Predicted SAT (Math + Verbal) score = 1351.71 + (−0.44 * World Rank), where World Rank can be found in Supplementary B.

**Table 2:** Relationship between sample size required for 80% statistical power, effect size of treatment, and correlation between covariate and outcome measure.

	Effect Size				
*ρ*	*d* = 0.1	*d* = 0.2	*d* = 0.3	*d* = 0.4	*d* = 0.5
0.0	3142	788	352	200	128
0.1	3111	780	348	198	127
0.3	2859	717	320	182	116
0.5	2357	591	264	150	96
0.7	1602	402	180	102	65

Note. *ρ* = correlation between covariate and dependent variable; All estimates are based on error rates of α = 0.05 and 1 − β = 0.80; Values in each cell represent the total number of participants to detect the specified effect size in a two-group randomized experiment with equal sample sizes in each group. In cases where the covariate is expected to be strongly related to the dependent variance (*ρ* = 0.5), including the covariate reduces the required sample size by roughly 25% (compare values in rows 1 and 4 of the table).

## Supplementary Materials

The following are available online at http://www.mdpi.com/2079-3200/6/3/37/s1. In Supplementary A, one asterisk (*) next to the school’s name indicates that at least some students were not required to supply scores to the school during the admissions process; two asterisks (**) indicates that the school did not report scores based on all students it had scores for, or did not tell U.S. News if it had; three asterisks (***) indicates that the data was reported to U.S. News from a previous year; and a tilde (~) indicates that the school “may not require scores from all applicants and may not have submitted data for all students.” For each of the schools with one of these qualifications, perhaps with the possible exception of (***), the ranking of the school is likely higher than it should be because average ability scores are overestimated. Regarding exception (*), if some students are not required to supply scores, it is typically students with lower scores who fail to report them, which allows schools to report higher SAT or ACT score averages (see [79]). Based on Wainer’s [79] analysis, exceptions (**) and (~) also would probably mean the average test scores reported are higher than if the school had reported average scores based on all students. This means schools with these exceptions should likely be lower in the current rankings than they presently are, but because it is unclear how much lower, they are still ranked based on the scores they reported for the purposes of this study. Supplementary B is a full list of schools that appeared in the 2018 *Times Higher Education* world ranking to enable researchers to utilize this along with Table 1 for interpolating the average SAT score or ability level of the student body of a non-U.S. school.
